# Manual therapy compared with physical therapy in patients with non-specific neck pain: a randomized controlled trial

**DOI:** 10.1186/s12998-017-0141-3

**Published:** 2017-04-28

**Authors:** Ruud Groeneweg, Luite van Assen, Hans Kropman, Huco Leopold, Jan Mulder, Bouwien C. M. Smits-Engelsman, Raymond W J. G. Ostelo, Rob A. B. Oostendorp, Maurits W. van Tulder

**Affiliations:** 1 Radboud University Nijmegen Medical Centre, Scientific Institute for Quality of Health Care, Geert Grooteplein 21, 6525 EX Nijmegen, The Netherlands; 2 Department of Health Sciences, Faculty of Earth & Life Sciences, Vrije Universiteit Amsterdam, Amsterdam Public Health research institute, De Boelelaan 1085, 1081 HV Amsterdam, The Netherlands; 3 Avans+, University of Applied Science, Heerbaan 14-40, 4817 NL Breda, The Netherlands; 4 Department of Health and Rehabilitation Sciences, University of Cape Town, Anzio Road 7935, Cape Town, South Africa; 5 Department of Epidemiology and Biostatistics & EMGO Institute for Health and Care Research, VU University Medical Centre, Amsterdam, The Netherlands; 6 Department of Manual Therapy, Vrije Universiteit Brussel, Faculty of Medicine and Pharmacy, Brussels, Belgium

**Keywords:** Randomized controlled trial, Neck pain, Manual therapy, Physical therapy, Effectiveness

## Abstract

**Background:**

Manual therapy according to the School of Manual Therapy Utrecht (MTU) is a specific type of passive manual joint mobilization. MTU has not yet been systematically compared to other manual therapies and physical therapy. In this study the effectiveness of MTU is compared to physical therapy, particularly active exercise therapy (PT) in patients with non-specific neck pain.

**Methods:**

Patients neck pain, aged between 18–70 years, were included in a pragmatic randomized controlled trial with a one-year follow-up. Primary outcome measures were global perceived effect and functioning (Neck Disability Index), the secondary outcome was pain intensity (Numeric Rating Scale for Pain). Outcomes were measured at 3, 7, 13, 26 and 52 weeks. Multilevel analyses (intention-to-treat) were the primary analyses for overall between-group differences. Additional to the primary and secondary outcomes the number of treatment sessions of the MTU group and PT group was analyzed. Data were collected from September 2008 to February 2011.

**Results:**

A total of 181 patients were included. Multilevel analyses showed no statistically significant overall differences at one year between the MTU and PT groups on any of the primary and secondary outcomes. The MTU group showed significantly lower treatment sessions compared to the PT group (respectively 3.1 vs. 5.9 after 7 weeks; 6.1 vs. 10.0 after 52 weeks).

**Conclusions:**

Patients with neck pain improved in both groups without statistical significantly or clinically relevant differences between the MTU and PT groups during one-year follow-up.

**Trial registration:**

ClinicalTrials.gov Identifier: NCT00713843.

## Background

In terms of number of years lived with disability, back and neck pain are most important condition worldwide [1] and usually runs an episodic course over a person’s lifetime [2–4]. Neck pain is an important individual, social and economic health problem, affecting up to two-thirds of adults at some point in their lives [3]. In the Netherlands, neck pain is the third most frequently self-reported musculoskeletal pain problem [5], Although many neck pain sufferers do not consult a health professional [6], the prevalence and costs of neck pain in primary care are high [7, 8]. Furthermore, neck pain has a major effect on participation, activities, work disabilities and is consequently associated with high indirect costs [3, 5, 9].

In cases of acute neck pain, general practitioners (GP) often take no immediate action [10]. If complaints persist for six months or longer, average perceived discomfort appears to remain fairly stable [11]. However, it is both clinically and economically important to ensure that patients do not enter a chronic phase. Evidence regarding treatment efficacy for sub-acute and chronic neck pain is currently inconclusive [12].

Manual therapy is a commonly used treatment for neck pain. Cochrane Reviews have shown that both manual therapy [12] and exercise therapy [13] are effective in the treatment of patients with neck pain. Studies in the Netherlands [14–16] in patients with sub-acute and chronic neck pain has shown significant differences in effectiveness and cost-effectiveness in favor of manual therapy compared with exercise therapy or usual GP care, both in short and long-term follow-up. Manual therapy according to the School of Manual Therapy Utrecht (MTU) is a specific form of spinal manipulative therapy in the Netherlands that differs in theoretical assumptions and principles from other manual therapies [17, 18]. In general, most manual therapies focus primarily on patient’s symptoms, particularly the main complaint, and on joint function and stability, range of movement, and the severity of symptoms [18]. MTU, in contrast, is guided by an assessment of preferred movement patterns of the individual patient and is performed by applying passive articular movements to all spinal and pelvic joints and all joints of extremities, with the goal of optimizing individual movement patterns. Treatment techniques used in MTU are based on arthrokinematic and osteokinematic principles and are comparable with the mobilization techniques used in other manual therapies. The main difference between MTU and other manual therapies and physical therapy is the assessment and treatment of the complete chain of joints of the spine, pelvis and extremities, independently of patient’s complaints, based on analysis of the individual movement pattern. Examples of preferred movement patterns are hand clasping, arm folding, and dominance of arm, leg and eye.

To date, no study has evaluated the effectiveness of MTU. Therefore, the objective of this study was to compare the short-term (7 weeks) and long-term effectiveness (52 weeks) of passive mobilization of the joint chain per MTU with active exercise therapy as usual care for physical therapy. The assumption was that MTU was more effective because of the above-mentioned characteristics of MTU. Patients with neck pain (more than two weeks and no longer than one year) were assessed regarding global perceived effect, functioning and pain.

## Methods

Methods have been described in detail elsewhere, and will be summarized below [17].

### Design overview

A pragmatic randomized controlled trial (RCT) was performed from September 2008 to February 2011.

### Settings and participants

Sixteen primary care practices participated in the trial. The manual therapists were enrolled via invitation by the research team and by collective invitation to the members of the professional association of the School of MTU. Participating manual therapists were asked to invite physical therapists (performing the active exercise therapy) with whom they collaborate in this study. Each participating practice had at least one manual therapist, one physical therapist and one research assistant. The latter were trained to perform the intake in a face-to-face meeting and with a video application. Twenty research assistants performed clinical tests to assess inclusion and exclusion criteria, and carried out history taking at baseline. Seventeen manual therapists and 27 physical therapists were involved in the study. Manual therapists had followed a three-year postgraduate program at the School of MTU following their physical therapy education. All participating therapists had a minimum of five years’ work experience.

Inclusion criteria for patient participation were: age 18 to 70 years; neck pain of any severity (cervical region from superior nuchal line to spine of scapula and superior border of the clavicula [4]) with or without radiation to the shoulder region or the upper extremity, with or without headaches; neck pain as main complaint for more than two weeks [4] and no longer than one year (inclusion of patients without spontaneous recovery within two weeks and without typical characteristics of long-lasting chronic neck pain, because these groups may benefit less from manual therapy); provocation or reproduction of pain by neck movement or neck and head posture.

Exclusion criteria were: presence of red flags, myelopathy, surgery of the cervical spine [19]; neck pain with a radicular pain pattern; entrapment neuropathy; pregnancy; whiplash injury (as cause of the complaint); any physical treatment for neck pain in the previous three months.

Patients entered the study either through direct access to a primary care practice or by GP referral, according to the Dutch healthcare system. Some patients were also recruited through articles in local newspapers. After signing informed consent, randomization took place.

### Randomization and interventions

Block randomization (block size of 4) was performed, pre-stratified for main complaint on numeric rating scale for pain (NRS-P) (range 0 (no pain) -10 (maximum pain)) (<7 or ≥ 7) and age (<40 or ≥ 40 years) [20, 21]. Independent research assistants, blinded for patient characteristics, allocated patients to one of the intervention groups using a central computer-generated randomization scheme.

### Manual therapy

MTU assessment is based on theoretical concepts of mechanobiology [22], and is described in the design article of this study [17]. General assessment includes history taking, screening for red flags, physical examination and treatment indication by the manual therapist. In addition, specific tests are used to evaluate the individual’s preferred movement pattern [23]. Examples of preferred movement patterns are hand clasping, arm folding, and dominance of arm, leg and eye. These tests have a high reliability (Kappa 0.8-1) [24].

The complete chain of joints of the spine, pelvis and extremities are mobilized whereby the direction of mobilization is theoretically determined by analysis of the tests of the individual preferred movement patterns. The manual therapist performs per protocol repeated passive joint movements with low velocity and intensity and high accuracy in different positions of the patient (sitting, supine and side-lying). The rhythm of the movements is slow (approximately 30 cycles/min) and the movements are repeated about six times. Treatment is in general painless. Passive joint movements are performed in a combination of rolling and sliding, or rocking and gliding (or swinging and sliding) in the joint, based on the arthrokinematic and osteokinematic principles of intra-articular movements. Passive movements are performed over the entire range of motion within the physiological range of motion of joints, whereby the curvature of the articular surface is followed, with manual forces directed to the joints/specific spinal level. Physiological joint range of motion is carefully respected. Traction, oscillation and high-velocity movements are not applied. In all patients, based on the assessment protocols, all joints of the spine, pelvis and extremities are mobilized in specific directions. The kind of the joint mobilization used is probably best comparable to grade III mobilization according to the principles of Maitland [25]. It is common to give advice on activities of daily living and lifestyle, and to recommend home exercises, customized to the patient as assessed by the manual therapist.

A treatment session takes 30 to 60 min. Treatment is repeated after one or two weeks. The maximum number of sessions is six over a six-week treatment period, determined per patient by the manual therapist and depending on the condition of the patient and/or progression of patient’s condition.

### Physical therapy

Prior to patient enrollment the participating physical therapists met and reached consensus on the treatment protocol. At the first appointment, the physical therapist enquired about history taking and physical examination. Treatments could consist of active exercise therapy, manual traction, muscle stretching and massage [26–28]. Manual mobilization techniques of the neck were not allowed. The aim of active exercises was to improve strength (particularly strengthening of the deep neck muscles and shoulder muscles), mobility of the neck, and movement coordination. Tailoring treatment was left to the discretion of the therapists and was based on a patient’s individual abilities, tolerance, condition and activities of daily living. The intensity level of the treatment was not defined.

Treatment sessions took place no more than twice a week, with a maximum of nine sessions. Session duration was approximately 30 min, determined per patient by the therapist. In each session, the physical therapist spent a minimum of twenty minutes on active exercise therapy combined with instruction. Advice on activities of daily living and lifestyle was also commonly offered to patients.

All participating manual therapists and physical therapists received a three-hour instruction session regarding interpretation and application of the study protocol. Deviations from the study protocol were registered, as were continuation of the treatment and co-interventions.

#### Co-interventions

During the intervention period, participating patients were asked not to use any other treatment besides those allocated, except for medication. Patients were free to use medication prescribed either by a physician or over-the-counter.

### Outcomes and follow-up

The patients completed questionnaires online or as a hard copy at baseline, 3, 7, 13, 26 and 52 weeks [17].

#### Prognostic factors

At baseline, demographic data, complaints and known prognostic factors were checked by history taking and questionnaires (Table 1). The Credibility/Expectancy Questionnaire (CEQ) [29] was completed, on a hard copy blinded for the therapist, after the first treatment session because expectations can influence treatment outcomes [30, 31]. The CEQ has been shown to be sufficiently valid and reliable [30]. Fear avoidance, measured with the Fear Avoidance Belief Questionnaire (FABQ) [32], is a risk factor for chronic pain and disability [31, 33, 34] and can predict outcomes [35, 36]. The general health questionnaire (SF36) was used to obtain a detailed health profile. The eight domains of the SF36 can be summarized into physical and mental component scores. The Dutch translation showed satisfactory validity and reliability (0.66 to 0.90, mean 0.84) [37]. Prior to randomization at baseline the research assistant verbally asked patients, and notes, if they had preferences to be treated with MTU or PT.

#### Primary outcomes

Global perceived effect (GPE) was measured using a 7-point ordinal scale (ranging from ‘much worse’ (1 point) to ‘complete recovery’ (7 points)). The GPE was dichotomized in responders (patients with ’complete recovery’ (7 points) and ‘much improved’ (6 points)) and non-responders (‘slightly improved’, ‘no change’, ‘slightly worse’, ‘much worse’, ‘worse than ever’). The GPE measures patient subjective global improvement and has a high face validity [38, 39] and an excellent test-retest reliability (ICC values of 0.90 to 0.99) [40].

Functioning was measured using the Neck Disability Index (NDI) [41]. The range of scores is 0–50; the higher the score, the greater the limitations in activities. The validity of the NDI is good [41], and reproducibility [42, 43] and responsiveness are acceptable [42–44]. The minimal clinically-important change (MCIC) on the NDI is 3.5 points [42, 44].

#### Secondary outcomes

Secondary outcomes were chosen to cover all domains of the International Classification of Functioning, Disability and Health (ICF) [45]. The NRS-P (range 0 = no pain to 10 = maximum pain) was used to assess neck pain intensity in the previous week. The NRS is a valid and responsive scale [46]. The MCIC on the NRS-P in patients with neck pain is 2 points [47].

Patients also registered severity, time of onset, duration and type of adverse events, when relevant, at 3, 7 and 13 weeks [48].

Additional the number of treatment sessions of the MTU group and PT group was analyzed.

### Sample size

Sample size calculation was based on both primary outcomes (GPE and NDI), whereby the GPE was chosen because this outcome variable requires the largest group of participants. A clinically-relevant difference of 20% in GPE was chosen, based on previous studies [14, 49]. Based on an α of .05 and 80% power (*β* = 0.2), 76 participants per intervention group were required. Considering a dropout rate of 15%, the aim was to include 90 participants in each group.

### Statistical analysis

The analyses were performed according to the intention-to-treat principle. A multilevel model was used to determine the effectiveness of the interventions over the follow-up period. In the analysis, patients were nested by practices Linear multilevel analyses were used for continuous variables and logistic multilevel analyses for dichotomous variables. All patients were analyzed. In multilevel analysis missing scores do not need an imputation strategy, as this type of analysis is very flexible in handling missing data [50]. As a secondary analysis, and following the intended analysis described in our design article [17], the differences between groups were tested by the Chi-Square test for dichotomous variables, and by ANOVA/mixed model (continuous variables), with Bonferroni post-hoc tests. A responder analysis was carried out on GPE and the NDI with a MCIC of ≥ 3.5 points [42] and for NRS-P on ≥ 2 points [47].

For evaluation of adverse events and patient preferences, Chi-square tests for categorical data and ANOVA, with Bonferroni post-hoc tests were used for continuous data. An assistant, blinded for patient details, handled all data registration. SPSS statistical software version 23 was used. For all comparisons, *P* ≤ 0.05 was considered statistically significant (two-sided). Missing values less than 15% were considered as acceptable, as suggested by PEDRO [51].

## Results

### Patient characteristics and baseline similarities

**Fig. 1:**
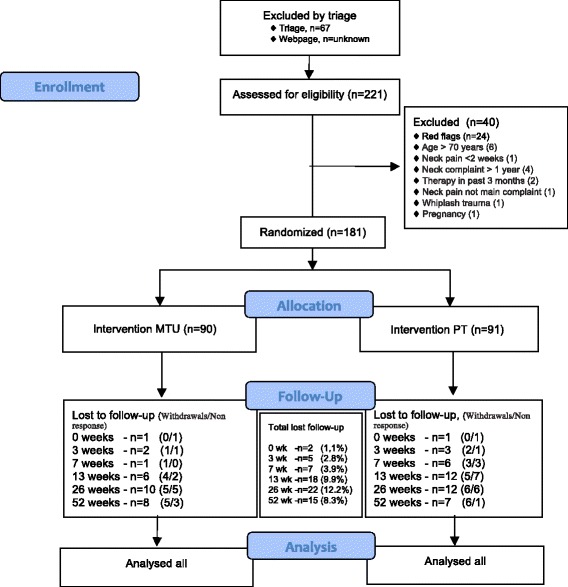
Flowchart of participation in the NECK project trial

A total of 221 patients were eligible, of whom 181 were randomized, ranging from 2-41 over the practices (see flow chart, Fig. 1, for more details). Mean patient age was 49 years (SD = 12.5), and approximately 55% were women. Most patients (57%) had neck complaints for the first time and most patients had multiple musculoskeletal complaints (60%). There were no important differences at baseline between the groups (Table 1).

**Table 1:** Baseline characteristics of the MTU group (*n* = 90) and PT group (*n* = 91)

Variable	MTU (*n* = 90)	PT (*n* = 91)
Age (in years, mean; SD)	49.2 (12.4)	48.7 (12.6)
Gender female (n; %)	56 (62.2)	56 (61.5)
First time neck complaints (n; %)	56 (62.2)	58 (63.7)
3 or more musculoskeletal complaints	54 (60.0)	55 (59.8)
4 or more musculoskeletal complaints	27 (30.0)	26 (28.3)
Combined neck and back pain	15 (16.7)	16 (17.4)
Last Year visit GP for neck complaints (n; %)	23 (25.6)	26 (28.6)
Last Year visit any therapist for neck (n; %)	17 (18.9)	20 (22.0)
Main complaints (n; %)		
Pain	79 (87.8)	80 (87.9)
Stiffness	30 (33.3)	38 (41.8)
Mobility impairment	33 (36.7)	37 (40.7)
Other	7 (7.8)	10 (11.0)
Complain intensity (NRS 0–10)		
Main complaint (mean; SD)	6.9 (1.3)	6.8 (1.4)
Second complaint (mean; SD)	6.0 (1.6)	5.9 (1.8)
Third complaint (mean; SD)	5.1 (2.1)	5.3 (2.0)
NDI (0–50) (mean; SD)		
	12.5 (6.8)	11.7 (5.4)
NRS pain (0–10) (mean; SD)		
	5.5 (2.0)	5.8 (1.8)
FABQ (mean; SD)		
Total (0–96)	30.0 (16.4)	29.6 (15.1)
Subscale work (0–66)	14.7 (13.5)	14.8 (12.3)
Subscale physical activity (0–30)	15.3 (5.4)	14.8 (5.7)
SF-36 (mean; SD)		
Physical component summary (0–100)	44.6 (7.9)	44.6 (7.1)
Mental component summary (0–100)	46.4 (12.5)	47.2 (10.0)
CEQ (mean; SD)		
Credibility (0–27)	22.0 (3.4)	22.4 (3.2)
Expectancy (0–27)	21.9 (4.8)	22.1 (4.7)
Intervention preference (n; %)		
None	61 (67.8)	63 (69.2)
Manual therapy	20 (22.2)	20 (22.0)
Physical therapy	9 (10.0)	8 (8.8)
Pre-stratification		
A. NRS main complaint ≤ 6, age ≤ 39	5	6
B. NRS main complaint ≤ 6, age ≥ 40	23	23
C. NRS main complaint ≥ 7, age ≤ 39	16	16
D. NRS main complaint ≥ 7, age ≥ 40	46	46

*Abbreviations*: *MTU* Manual Therapy Utrecht, *PT* Physical Therapy, *SD* Standard Deviation, *NRS pain* Numeric Rating Scale, *NDI* Neck Disability Index, *FABQ* Fear Avoidance Belief Questionnaire, *SF-36* Short Form-36, *CEQ* Credibility/Expectancy Questionnaire, *SD* Standard deviation

### (Intention-to-treat) analysis

**Fig. 2:**
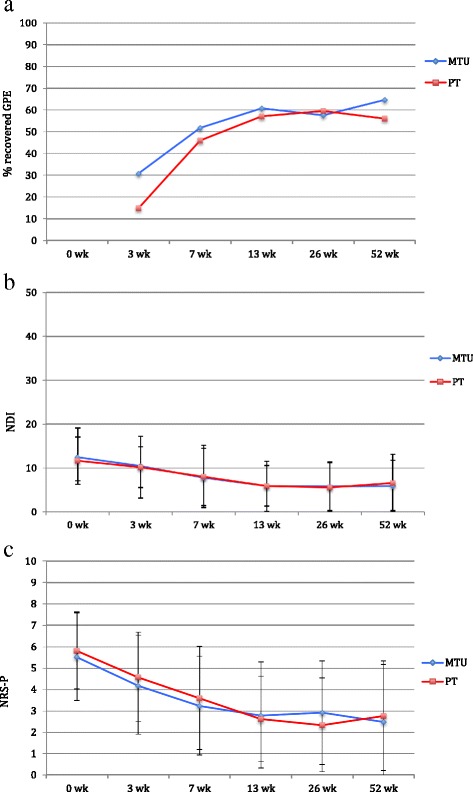
Scores, with standard deviation bars, for manual therapy (MTU group; *n* = 90) and physical therapy (PT group; *n* = 91): **a** – GPE: Global Perceiver Effect (% recovery), **b** – NDI: Neck Disability Index (0-50), **c** – NRS-P: Numeric Rating Scale for Pain (0–10). *Abbreviations*: *GPE* Global Perceived Effect, *NDI* Neck Disability Index, *NRS-P* Numeric Rating Scale for Pain, *wk*. weeks, *MTU* Manual Therapy Utrecht, *PT* Physical Therapy

Missing values accounted for 3.9% at 7 weeks and 8.3% at 52 weeks. In multilevel analyses, there were no statistically significant overall differences at one year between the two groups on the primary and secondary outcomes GPE, NDI, or NRS-P (Table 2, Fig. 2). GPE showed statistically significantly higher scores only at 3 weeks in favor of the MTU group; adjusted odds ratio 3.97 (95% confidence interval [CI], 1.31 to 12.04; *P* = 0.02). Pain intensity scores were lower for the PT group only at 26 weeks; mean adjusted differences of 0.78 points (95% CI, 0.09 to 1.47, 95% CI; *P* = 0.03). There were no other statistically significant differences. ANOVA for NDI and NRS-P, and Chi-square tests for GPE, showed identical results.

**Table 2:** Mean scores, regression coefficients, and odds ratios for primary and secondary outcomes, and responder analyses (Intention to Treat, *N* = 181)

Variables	Manual therapy Utrecht (*n* = 90)	Physiotherapy (*n* = 91)	Regression coefficients (95% CI)	
Primary outcomes measures				
GPE (success, %)			OR (95% CI)	*P*
Overall effect: *P* = 0.20				
3 weeks.	30.7	14.8	3.97 (1.31 to 12.04)	0.02**
7 weeks.	51.7	42.4	1.90 0.69 to 5.20)	0.21
13 weeks.	60.7	57.0	1.11 (0.39 to 3.15)	0.84
26 weeks.	57.5	59.5	0.76 (0.26 to 2.18)	0.61
52 weeks.	64.9	56.0	1.72 (0.61to 4.88)	0.31
NDI (0–50) (M, SD)			Regression Coefficients (95% CI)	*P*
Overall effect: *P* =0.57				
Baseline	12.5 (6.8)	11.7 (5.4)		
3 weeks.	10.5 (6.9)	10.2 (5.6)	−0.24 (−1.65 to 1.16)	0.74
7 weeks.	7.8 (6.4)	8.1 (7.1)	−0.69 (−2.14 to 0.76)	0.35
13 weeks.	5.9 (5.8)	5.9 (4.6)	−0.23 (−1.67 to 1.21)	0.75
26 weeks.	5.9 (5.5)	5.6 (5.6)	0.34 (−1.11 to 1.80)	0.64
52 weeks.	5.9 (5.7)	6.6 (6.5)	−0.82 (−2.25 to 0.61)	0.26
Secondary outcomes measures				
NRS-P (0–10) (M, SD)				
Overall effect: *P* = 0.76				
Baseline	5.5 (2.0)	5.8 (1.8)		
3 weeks.	4.2 (2.3)	4.6 (2.1)	−0.27 (−0.94 to 0.40)	0.42
7 weeks.	3.2 (2.3)	3.6 (2.4)	−0.20 (−0.87 to 0.47)	0.56
13 weeks.	2.8 (2.5)	2.6 (2.0)	0.24 (−0.44 to 0.93)	0.49
26 weeks.	2.9 (2.4)	2.3 (2.2)	0.78 (0.09 to 1.47)	0.03**
52 weeks.	2.5 (2.6)	2.8 (2.6)	−1.14 (−0.82 to 0.54)	0.69
Responder analyses				
MCID NDI (success, %)			OR (95% CI)	*P*
Overall effect: *P* = 0.95				
3 weeks.	31.0	20.7	2.08 (0.79 to 5.43)	0.14
7 weeks.	56.3	52.0	1.26 (0.49 to 3.24)	0.63
13 weeks.	66.3	65.8	0.97 (0.37 to 2.53)	0.95
26 weeks.	62.0	69.6	0.55 (0.21 to 1.47)	0.23
52 weeks.	60.5	63.1	0,79 (0.31 to 2.02)	0.63
MCID NRS-P (success, %)				
Overall effect: *P* = 0.11				
3 weeks.	34.9	37.2	0.87 (0.38 to 2.14)	0.73
7 weeks.	55.2	56.6	0.95 (0.42 to 2.14)	0.89
13 weeks.	59.4	65.2	0.56 (0.23 to 1.38)	0.21
26 weeks.	58.2	79.5	0.25 (0.10 to 0.63)	0.00**
52 weeks.	69.1	69.9	0.93 (0.39 to 2.25)	0.88

Values presented are model estimates of general linear mixed-effects models with a random intercept and adjusted for baseline and location. Regression coefficients can be interpreted as mean differences between interventions at a certain follow-up moment compared with baseline. Positive values favor the manual therapy group For Odds Ratios the reference group is physiotherapy

*Abbreviations*: *ITT* intention-to-treat, *CI* confidence interval, *GPE* global perceived effect, *NDI* Neck Disability Index, *NRS-P* Numerical Rating Scale for Pain, *OR* odds ratio, *MCID* Minimal Clinical Important Difference

Responder analysis is presented in Table 2. Overall, no statistically significant differences were found except for GPE at 3 weeks (adjusted odds ratio 3.97, see above), and for pain at 26 weeks (NRS-P ≥2 points) in favor of the PT group (80%) versus the MTU group (58%); adjusted odds ratio of 0.25 (95% CI, 0.10 to 0.63; *P* = 0.00).

The MTU group participated in a mean of 3.1 (SD = 1.5, range 0–8) treatment sessions during the intervention period versus 5.9 (SD = 3.4, range 0–20) for the PT group (7 weeks). Over the one-year follow-up period the number of treatments was 6.1 in the MTU group (SD = 5.4, range 0–33) versus 10.0 (SD = 6.8, range 0–29) in the PT group.

### Adverse events

Minor, short-term adverse events were reported, but no serious complications occurred (Table 3). There were no significant differences in adverse events between the two groups in number (*n* = 40 for MTU group and *n* = 31 for PT group), severity (NRS-P 4.8 for MTU group and 5.4 for PT group) or duration (mostly less than 24 h). The most frequently reported reactions were increased neck pain (17 out of 40 in MTU group and 14 out of 31 PT group), headache (3 of 40 in MTU group and 5 of 31 in PT group), back pain (7 of 40 in MTU group, 1 out of 31 in PT group), and dizziness and pins and needles in arms (2 in both groups).

**Table 3:** Adverse events for both treatments

Adverse events		3 weeks			7 weeks		13 weeks		
	MTU	PT	p	MTU	PT	p	MTU	PT	p
Number	40	31	0.1723	31	23	0.1964	17	12	0.3179
Severity (NRS)	4.8 (3.9)	5.3 (2.6)	0.3607	4.1 (3.1)	3.8 (2.8)	0.6456	4.9 (3.0)	3.6 (2.8)	0.2508
Time of onset (n)									
< 30 min.	4	7		5	4		3	0	
>30 min. < 4 h.	14	7		5	4		2	3	
> 4 h., < 24 h.	11	8		11	8		3	1	
> 24 h.	7	6		5	5		6	1	
Duration (n)									
< 10 min.	0	0		3	2		4	1	
> 10 min., < 1 h.	4	5		1	2		2	0	
> 1 h., < 24 h.	13	9		10	5		8	3	
> 24 h.	20	13		13	12		4	2	

*Abbreviations*: *MTU* Manual Therapy Utrecht, *PT* Physical Therapy, *NRS* Numeric Rating Scale, *n* number, *min* minutes, *hr* hours

### Patient preferences

In both groups 20 patients preferred MTU. In the MTU group 9 patients preferred physical therapy, whereas 8 patients in the PT group preferred physical therapy. Other patients had no treatment preference. No differences on the mean CEQ scores were found between the MTU group and the PT group and between the patients preferring MTU or PT, compared to the non-preference patients. No significant differences in GPE, functioning (NDI) or pain (NRS-P) were found between patients who received their preferred intervention (*n* = 28) compared with those who did not receive their preferred treatment (*n* = 29).

## Discussion

This pragmatic RCT in 181 patients with non-specific neck pain (>2 weeks and <1 year) found no statistically significant overall differences in primary and secondary outcomes between the MTU group and PT group. The results at 7 weeks and 1 year showed no statistically and clinically significant differences. The assumption was that MTU was more effective based on the theoretical principles of mobilization of the chain of skeletal and movement-related joint functions of the spine, pelvis and extremities, and preferred movement pattern in the execution of a task or action by an individual, but that was not confirmed compared with standard care (PT).

### Adverse events

Around 40% the patients in total reported various minor adverse events attributable to the treatment, following the classification terms for adverse events by Carnes et al. [48]. There were no significant differences between the MTU group and PT group in number, severity or duration of adverse events. RCTs are not the best research method for estimating the frequency of adverse events due to the recorded design of inclusion and treatment protocol. Large observational cohort studies might be expected to give a more accurate assessment [52].

### Attention bias and treatment sessions

In this study, the mean number of treatment sessions in the MTU group was lower at 7 weeks and at 1 year. The initial maximum number of treatment sessions was 6 for MTU and 9 for PT; the time per session was 30-60 min and 30 min for MTU and PT, respectively. These parameters were described in the study protocol and are in line with standard Dutch clinical practice. Although the registration of the duration (number of minutes) of each treatment session was incomplete, the available data showed that only a minority (10%) of patients in the MTU group was treated longer than 30 min. So, overall the number of treatment sessions was higher in the PT group. However, as there were no differences in effects, we consider the risk of attention bias to be low.

### Cost-effectiveness

A review of Michaleff et al. [53] supported the use of spinal manipulative therapy (SMT) in clinical practice as a cost-effective treatment when used alone or in combination with other treatment approaches. This review showed that while the effectiveness of SMT is comparable to other treatments, SMT is the more cost-effective treatment option. As Rubinstein et al. [54] have stated, because many treatments for spinal pain have comparable outcomes, distinctions in terms of cost-effectiveness should enjoy a high priority. An evaluation of the cost-effectiveness of the present study was recently published [55]. In summary, the intervention costs and healthcare costs were significantly lower in the MTU group than in the PT group, whereas unpaid productivity costs were significantly higher. Total costs did not significantly differ between the MTU group and PT group. The conclusion was that MTU was not cost-effective in comparison with PT [55]. Consequently, the choice for MTU or PT can be left to the preferences of patients and care providers.

### Perspectives in relation to literature

The patient population in our pragmatic study is comparable to populations described in general medical care. Additionally, the prevalence of co-morbidity is in line with the prevalence of co-morbidities for people visiting medical practices [56].

Our results are in line with the international literature on this subject, suggesting that SMT has a similar efficacy to other treatments [12, 57–60].

Present results are probably most comparable to two other Dutch studies by Hoving et al. [14, 15] and Pool et al. [61], given similar patient populations within the Dutch health care system. This is evident from the comparison of baseline scores (i.e. pain, function, duration of symptoms) with those studies [14, 15, 61]. Hoving et al. [14, 15] compared manual therapy to physical therapy and usual care (mainly based on a “wait and see” policy) provided by GPs. Manual therapy and PT were found to be more effective than GP care, with manual therapy superior to physical therapy, in contrast to our study. The reasons behind these differences of these studies may include slightly older participants in our study (mean age 50 years) compared to Hoving et al. and Pool et al (both mean age 45 years) [14, 15, 61]. A second difference was the higher success rate on GPE in the studies of Hoving et al. (72% in MT group) [14, 15] and Pool et al. (70% in MT group) [61], compared to this study (61% in MTU group) at 13 weeks. This may have been due to the way data on outcomes were collected. In the study by Hoving et al. [14, 15] patients visited the research assistant and in the study by Pool et al. [61] a researcher collected patient data during a home visit. Because patients participating in research are known to try to meet an investigator’s expectations [62], we eliminated personal contact between patients and research assistant through the use of web-based questionnaires and by avoiding face-to-face meetings or other personal contact during the follow-up period. A third explanation may be differences between the interventions compared to Hoving et al. [14, 15]. While the aim of PT treatment was comparable in both studies, Hoving et al. [14, 15] used manual therapy based on coordination and stabilization techniques (exercises) to treat segmental movement dysfunction of the spine, which might explain a better outcome for the MT group compared to the PT group.

The aim of this study was to evaluate the effectiveness of MTU as monotherapy and to compare MTU with PT, particularly exercise therapy. Both monotherapies are equally effective in patients with neck pain. It appears that clinicians are more and more in favor of using a broad-spectrum approach to treating patients with musculoskeletal pain, particularly neck pain and low back pain. Manual therapy is often combined with exercise therapy and pain education to treat patients with neck pain. Moderate quality evidence supports this combined therapy [63]. Studies of multimodality therapy should consider appropriate study designs, such as factorial designs.

### Pattern of responses to primary care treatments

If the results of this study are placed in a broader perspective, a common trend in outcomes is visible, as shown in the pattern of treatments outcomes in patients with low back pain [12, 64]. After an initial improvement in pain and functioning for both groups within 13 weeks, a slower reduction and stabilization of pain and functioning were followed in up to 52-week follow-up in both groups. This pattern of treatment outcome is quite similar in clinical trials with patients with low back pain unrelated to the type of treatment, particularly manual therapies It is plausible that such a pattern of responses to primary care treatments also occurs in clinical trials with patients with neck pain [12].

Artus et al. [64] provide several explanations for these comparable treatments outcomes in trials. Firstly, non-specific factors, such as natural history and regression to the mean, non-specific effects of treatments (patients’ and therapists’ characteristics such as their beliefs, expectation, previous experiences and the attention given in the trial), differences of mean versus individual responses (averages in trials neutralizing individual variation) and the large overall response to treatment.

Besides these non-specific factors, there are intervention-specific factors. The treatment used in this trial was a manual therapeutic treatment per MTU that involves specific treatment ingredients based on arthrokinematic and osetokinematic principles of intra-articular joint movements of the spine, pelvis and extremities, as basis for promoting the individual preferred movement pattern.

With neck pain, many treatment options, particularly manual therapies, are available with often limited scientific evidence. Internationally, there are different schools of manual therapy. Some of these schools function as distinct professional groups (e.g., chiropractors, napropaths and osteopaths), while others consider themselves a specialization of physiotherapy (e.g., manual physical therapists). There is discussion about whether the treatment techniques used in these different schools contain essential differences and intervention-specific factors [18]. On the one hand, manual therapists, physiotherapists, chiropractors, napropaths, and osteopaths are educated in different theoretical assumptions and supposed underlying mechanisms, and on the other hand they use slightly different treatment techniques [18]. It has been suggested that the differences in practical applications of manual techniques are found mainly in amplitude and velocity of the mobilization and manipulation techniques [18]. However, it is questionable whether these schools are so different. There is room for discussion whether it is necessary and/or desirable emphasizing this diversity in manual therapies [65], considering that, despite the differences in approach (which are often small, subtle and theoretical in nature), there are no demonstrable differences in effectiveness [12].

### Limitations

Some weaknesses in this study should be considered when interpreting our results. One issue deals with recruitment of patients, which started well but later slowed down. We therefore added an additional recruitment strategy using local newspapers. This could potentially have influenced the type or the severity of symptoms of patients at baseline [66, 67]. It has been shown that the recruitment method affected the clinical characteristics (number of joints affected) and physical functioning (pain and tiredness) of patients recruited for a study of osteoarthritis of the hip or knee. However, a mix of recruitment strategies as used in our study should not affect treatment outcomes, on the condition that adjustments are made for differences at baseline [67–69]. The chosen strategy has no influence on the results.

A second issue worthwhile discussing is the partial overlap between the two treatment arms in this study. Although this is often unavoidable in a pragmatic trial, it may have resulted in bias. In both interventions patients received individualized advice with respect to activities of daily living and lifestyle and all received homework exercises. This may have decreased the contrast between the MTU group and the PT group, making this too small to lead to differences in outcomes.

A third potential limitation is the lack of a ‘no-treatment’ arm in this study. As it is not acceptable on ethical grounds to compare MTU with no treatment, because with no intervention patients are withheld a proved effective intervention. An indirect comparison with previous research in a similar setting is the most feasible approach to differentiate our results between regression to the mean and the natural history of the disease. Hoving et al. [14, 15] compared manual therapy and physical therapy with a ‘wait and see’ policy in Dutch primary care. The results of this study suggested that manual therapy and physical therapy are more effective than the “wait and see” policy. It seems reasonable to assume that the effects of MTU and PT identified in our study do not reflect the natural course or regression to the mean.

Another issue that needs to be taken into account is the mean baseline scores on NDI for both groups, which are low in our study, 12.5 in the MTU group and 11.7 in the PT group, equivalent to mild neck disability [70]. Kato et al. [71] determined the cut-off value of the NDI to detect neck pain associated with disability in a Japanese population to be 15 points. As this was a Japanese study, intercultural differences could play a role in the perceived limitations of activities. During follow-up, the NDI scores improved in both intervention groups. The mean scores at 7 weeks (7.9 points in both groups) and 13 weeks (5.9 points in both groups) were considered as ‘no remaining disability’ [70]. In this NDI range (around 12 of 50 points, SD +/- 6) statistically significant and clinically meaningful differences between groups are probably difficult to detect. The low baseline scores may explain why no clinically relevant and significant differences on the NDI scores were observed between the two groups, and there was potentially little room for improvement.

### Clinical implications

The results of this study are that MTU and PT (active exercise therapy) do not differ in terms of effectiveness with both groups achieving similar improvements in pain and functioning. In shared decision-making, the choice of treatment options will be based on personal preferences of patient and therapist, with previous patient’s and therapist’s experience and the expected number of treatments to play a role.

## Conclusions

Patients with non-specific neck pain improved in both groups without statistical significantly or clinically relevant differences between the MTU and PT groups during one-year follow-up.

## Abbreviations

MTU: Manual Therapy Utrecht
PT: Physical therapy
GP: General practitioners
RCT: Randomized controlled trial
NRS: PNumeric rating scale for pain
CEQ: Credibility/Expectancy Questionnaire
FABQ: Fear Avoidance Belief Questionnaire
GPE: Global perceived effect
NDI: Neck Disability Index
MCIC: Minimal clinically important change
ICF: International Classification of Functioning, Disability and Health
SMT: Spinal manipulative therapy
